# DEKODE—A cloud‐based performance feedback model improved DKA care across multiple hospitals in the UK

**DOI:** 10.1111/dme.70004

**Published:** 2025-02-17

**Authors:** Lakshmi N. Rengarajan, Catherine Cooper, Kashish Malhotra, Angelica Sharma, Nevil Philip, Anu Ann Abraham, Ketan Dhatariya, Parth Narendran, Punith Kempegowda, DEKODE and DEVI Group, Nabeel Ahmed, Lucy Bomphrey, Amy Birchenough, Shams Ali Baig, Joseph Dalzell, Meghna Hebbar, Saima Kauser‐Malik, Sophie Mounouchos, Arjun Narendran, Hariharan Paneerselvam, Dineshwaran Rajendran, Maria Skaria, Shamanth Soghal, Wai Nga Alice Yip, Jayashekara Acharya, Jason Cheung, Parijat De, Muhammad Ali Karamat, Senthil Kumar Krishnasamy, Rajeev Raghavan, Sanjay Saraf, Alexandra Solomon, Jael Nizza

**Affiliations:** ^1^ Department of Applied Health Sciences University of Birmingham Birmingham UK; ^2^ Queen Elizabeth Hospital University Hospitals Birmingham NHS Foundation Trust Birmingham UK; ^3^ Walsall Manor Hospital The Walsall Healthcare NHS Trust Walsall UK; ^4^ Rama Medical College Hospital and Research Centre Uttar Pradesh India; ^5^ School of Public Health, Faculty of Health and Medical Sciences University of Adelaide Adelaide South Australia Australia; ^6^ Norfolk and Norwich University Hospitals NHS Foundation Trust Norwich UK; ^7^ Institute of Immunology and Immunotherapy University of Birmingham Birmingham UK

**Keywords:** adherence to guidelines, diabetic ketoacidosis, glucose monitoring, health equity, quality improvement project

## Abstract

**Aim:**

A current gap in Diabetes‐related ketoacidosis (DKA) research is understanding the factors contributing to variations in care and outcomes between people admitted with DKA. We aimed to create a system to facilitate gathering data on DKA management across multiple centres and identify trends in complications and outcomes associated with DKA.

**Research Design and Methods:**

Between January 2020 and December 2022, we set up a cloud‐based Quality improvement project (QIP) that provided regular feedback to 11 hospitals in the United Kingdom (UK).

**Results:**

Of the 1977 episodes, we observed an increase in adherence in fluid prescription in hospitals C, D, E, F and G (C‐ 23% vs. 75% *p* = <0.001; D‐ 27% vs. 60%, *p* = <0.001; E‐ 17 vs. 79% *p* = <0.001; F‐ 16% vs. 57%, *p* = <0.001; G‐ 36% vs. 75% *p* = <0.001). Notable improvements in adherence to glucose monitoring were observed in hospitals B, D, and G (B‐ 11 vs. 38% *p* = <0.001; D‐ 36% vs. 56%, *p* = 0.05; G‐ 22% vs. 67% *p* = <0.001). Although we didn't observe significant changes in complications and outcomes among participating hospitals from the start to the end of the reported period, notable fluctuations were evident across quarters. These variations were relayed to the respective hospitals, underscoring how feedback and interventions could influence the care provided. This initiative also marks the initial move towards establishing and improving data collection practices in acute diabetes.

**Conclusions:**

We demonstrate a sustainable QIP that improves adherence to national guidelines in some indicators for DKA care and serves as an early warning system to identify adverse trends.

**What's new?**

**What is already known?**

- A current gap in Diabetes‐related ketoacidosis (DKA) research lies in understanding the factors contributing to variations in care and outcomes between people admitted with DKA.

**What this study has found?**

- Of the 1977 episodes, we observed an increase in adherence in fluid prescription in hospitals C, D, E, F and G (C‐ 23% vs. 75% *p* = <0.001; D‐ 27% vs. 60%, *p* = <0.001; E‐ 17 vs. 79% *p* = <0.001; F‐ 16% vs. 57%, *p* = <0.001; G‐ 36% vs. 75% p = <0.001). Notable improvements in adherence to glucose monitoring were observed in hospitals B, D, and G (B‐ 11 vs. 38% *p* = <0.001; D‐ 36% vs. 56%, *p* = 0.05; G‐ 22% vs. 67% *p* = <0.001).

**What are the implications of the study?**

- We demonstrate a sustainable QIP that improves adherence to national guidelines in some indicators for DKA care and serves as an early warning system to identify adverse trends.

## 1 INTRODUCTION

Diabetes‐related ketoacidosis (DKA) is one of the most common reasons for unplanned emergency hospital admission in people with diabetes.^1^ DKA is characterized by a triad of hyperglycaemia, ketosis and acidosis.^2^ Structured guidelines have transformed DKA from an inevitable fatality to a treatable complication.^3^, ^4^ While policies have improved DKA management, morbidity rates remain high.^5^, ^6^ Recent data shows a rising incidence of DKA in the UK, escalating healthcare costs.^6^, ^7^, ^8^ There were 32,920 DKA episodes in the UK from September 2021 to August 2022, costing the NHS over £90 million.^9^ In the United States, a review of national DKA admissions showed a three billion dollar increase in the aggregate bill for people admitted with DKA in the past two decades.^10^ A current gap in DKA research lies in understanding the factors contributing to variations in care and outcomes between people admitted with DKA.^11^ Identifying these crosslinks can offer insights that enable the development of targeted interventions, improve outcomes for people admitted with DKA, and promote equitable DKA care among diverse populations.

Audits are a common way to assess the quality of care,^12^ and when it is combined with feedback, it can be utilised to refine clinical practice.^13^ However, audits have limitations due to their time‐consuming and resource‐intensive nature, limited scope, and potential for bias.^14^, ^15^, ^16^ The development of bespoke audits by different hospitals results in duplication and hinders the sharing of best practices.

The National Diabetes Inpatient Safety Audit (NDISA) is a rolling national audit programme that collects information on four life‐threatening diabetes‐specific inpatient harms: hypoglycaemia, DKA, hyperosmolar hyperosmotic state (HHS), and diabetic foot ulcer (DFU). However, it only provides trends on the frequency of these complications. It does not provide information on the aetiology of DKA or the compliance rates with specific parameters of DKA management.^17^

Previously, we showed that a quality improvement programme (QIP) that provides regular stakeholder feedback on key performance indicators could sustain improvement in DKA care.^18^ Fixed‐rate intravenous insulin infusion (FRIII), fluid administration, and hourly monitoring of glucose and ketone measurements alongside management of complications like hypokalaemia, hypoglycaemia and hyperkalaemia formed this model's core elements of regular feedback.^3^, ^19^ The data from this QIP helped evaluate DKA management during the COVID‐19 pandemic^20^ and understand the changing trends in DKA and its management.^21^ Building on this, we hypothesised that a uniform system could minimise administrative and logistic duplication of work associated with monitoring DKA management across multiple centres. To ascertain this hypothesis, we designed this study with the following objectives:

- To establish a sustainable QIP across participating hospitals.
- To study the change in adherence trends to FRIII prescription, fluid administration, glucose and ketone monitoring per national guidelines in response to regular feedback.
- To study the changes in the trends of complications (hypoglycaemia, hypokalaemia, and hyperkalaemia) and outcomes (DKA duration and length of stay) of DKA management across time in response to regular feedback.

## 2 RESEARCH DESIGNS AND METHODS

### 2.1 Context

In 2020, we expanded the successful QIP‐based DKA management monitoring system to three hospitals, thus establishing the first cohort of the DEKODE (Digital Evaluation of Ketosis and Diabetes‐related Emergencies) model, a cloud‐based DKA management monitoring system. Two more hospitals (D and E) joined in 2021, and the other six (F to K) joined in 2022. All participating hospitals had a similar integrated care pathway for DKA management based on the Joint British Diabetes Societies‐Inpatient (JBDS‐IP) guidelines.^3^

### 2.2 Process mapping and interventions

Upon acceptance of an invitation to join the QIP, a team was established to manage the DKA management monitoring system in the interested hospital, led by a junior doctor or medical student and supervised by a consultant. The group registered the QIP with their local clinical governance department. After the local clinical governance team authorised to proceed to data collection, junior doctors liaised with the informatics department of their respective hospitals to obtain the list of people discharged with an ICD‐10 code for DKA (E10∙0, E11∙0, E12∙0, E13∙0, E14∙0, E10∙1, E11∙1, E12∙1 E13∙1, E14∙1). This list was combined with a list of those treated with FRIII to screen for DKA. This facilitated the inclusion of episodes not identified in the list generated by the local health informatics team. This occasionally happens if the episode is not coded as DKA through ICD‐10 codes. A local team member then manually confirmed if these episodes met the DKA diagnosis based on the JBDS‐IP guidelines.^3^ Once approved, these episodes were assigned a code. The team collected data on DKA management for these episodes on the Google form DEKODE. A list of patients included in the QIP with their identifiable information (name, hospital identification number, NHS number, Date of Birth) was retained in the local centre for future reference. Pseudonymised data of these DKA episodes were recorded and stored in a secure, centralised DEKODE platform accessible to key team members. Using pseudonymised code ensured no patient‐identifiable data was collected on the Google form. A local database matching the codes to appropriate DKA episodes meant we could review the data if needed after the entry. A visual representation of the DEKODE process is depicted in Figure S1.

**FIGURE 1:**
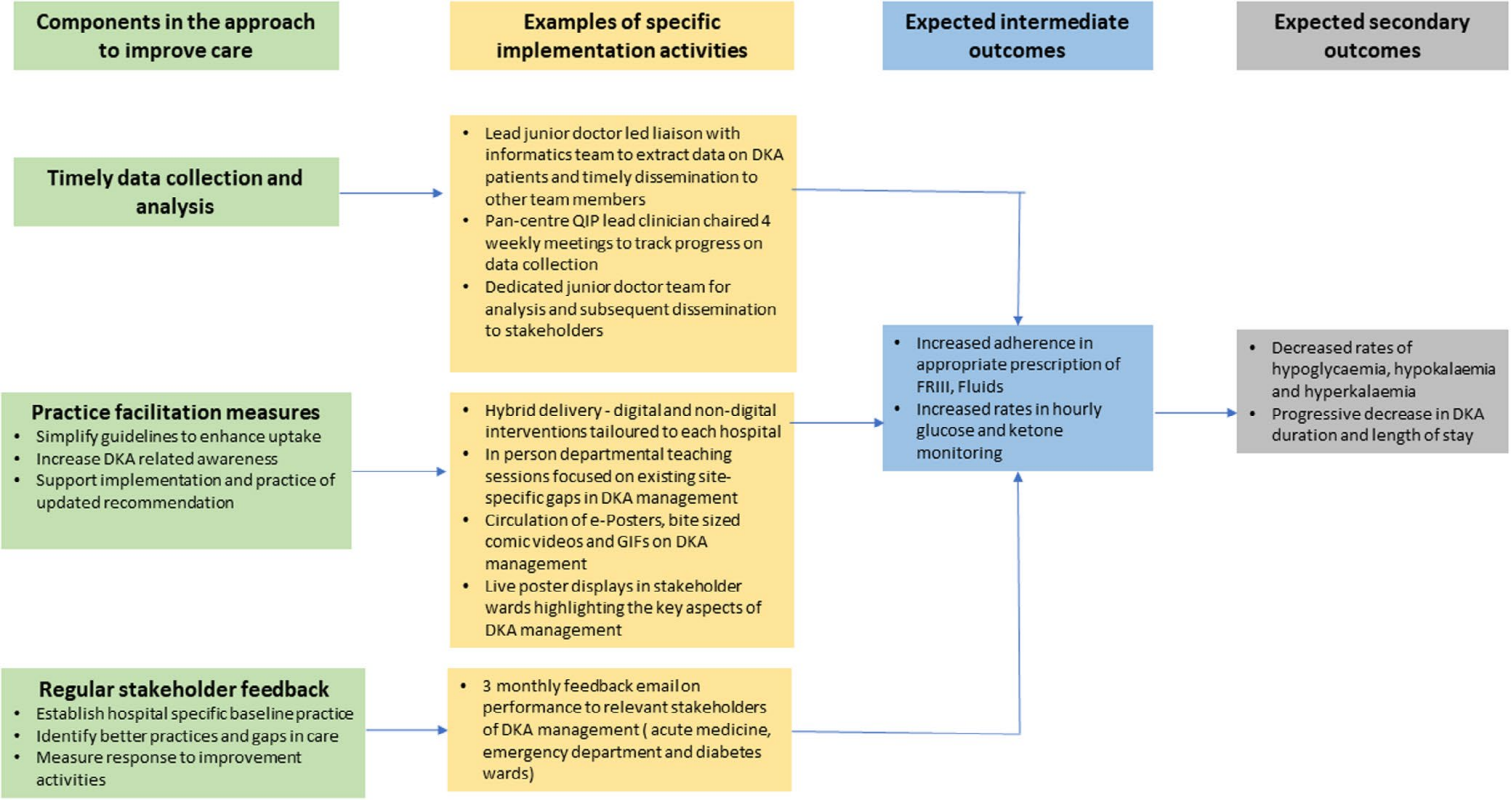
Comprehensive Framework for Improving DKA Care using DEKODE model: From Data Collection to Patient Outcomes.

Every 3 months, the data from DEKODE Google forms were analysed for key performance indicators (adherence to national recommendations for DKA care for FRIII, fluid administration, hourly monitoring of glucose and ketone measurements), complications (proportions of hypokalaemia, hypoglycaemia, and hyperkalaemia), and outcomes of DKA management (median and interquartile range (IQR) of DKA duration and length of stay for DKA episodes). The trends of these parameters over 12 months compared to the anonymised summative performance of all participating hospitals in the DEKODE model were provided to each participating hospital. Based on the input, participating hospitals were encouraged to implement various in‐person and virtual interventions to highlight areas of potential improvement based on DEKODE feedback. These interventions ranged from departmental meetings and teaching sessions to circulating posters about awareness of DKA and its management across departments. Virtual delivery included online teaching sessions and circulation of digital signs, bite‐sized concise videos on DKA to clinicians and allied health care professionals involved in DKA management. The timelines of various interventions across participating hospitals between January 2020 and December 2022 are depicted in Figure S2. Figure 1 outlines the process and practice facilitation measures to improve DKA management.

### 2.3 Measures

All DKA admissions aged ≥16 years from January 2020 to December 2022 across 11 hospitals were included for analysis. We defined DKA as per recommendations by the JBDS‐IP guidelines (capillary or serum glucose >11 mmol/L or a history of diabetes, and capillary or serum ketones >3 mmol/L or urine ketones ≥ ++ and pH <7.30 or bicarbonate <15 mmol).^3^ DKA resolution was defined as pH >7.30 or bicarbonate >18 mmol and capillary or serum ketones <0.6 mmol/L for two consecutive hours. Classification of the type of diabetes (type 1 [T1DM] or type 2 [T2DM]) was based on the clinical diagnosis documented in patient records. Alongside baseline sociodemographic, we recorded the following parameters for each DKA episode:

#### 2.3.1 Rate of FRIII administered

According to JBDS‐IP guidelines, FRIII should be started at 0.1 units/kg/h, and the patient's long‐acting insulin should be continued alongside. In the 2021 revision, JBDS‐IP recommended reducing the rate of FRIII to 0.05 units/kg/hour when the glucose falls below 14 mmol/L.^3^, ^22^ Adherence to FRIII in the percentage of JBDS‐IP recommendation was calculated using the formula: (FRIII/(Weight/10)) × 100. The patient's weight from the index admission was either estimated or manually recorded to facilitate this. For example, the recommendation for a 70 kg patient admitted with DKA is 7.0 units per hour, amounting to 100% adherence. If a prescription of 3.5 units/h or 14.0 units per hour is recorded for the same patient, adherence would be derived as 50% or 200%, respectively. Values below 100% imply adherence less than the recommended dosage, whilst values above 100% imply adherence more than the recommended dosage.

#### 2.3.2 The volume of fluids administered

The recommendation for fluid prescription during DKA is as follows: 500 mL bolus till systolic blood pressure (BP) >90 mmHg, then 1000 mL over 1 hour, 1000 mL over 2 h with potassium replacement (repeated twice), 1000 mL over 4 h with potassium replacement (repeated twice), followed by 1000 mL over 6 h with potassium replacement until DKA resolves.^3^ The total volume of fluids prescribed for the patient during their DKA episode was noted. The following formulae determined the adherence of fluids in percentage (Table S1). For example, if the DKA duration was 5 h, the recommendation is 4 L equating to 100% adherence. If a prescription of 3 or 5 L is recorded for the same patient, the adherence would be derived as 75% or 125%, respectively.

Frequency of glucose and ketone monitoring: Adherence to glucose and ketone monitoring per cent was calculated using the formula (the total number of readings/DKA duration in hours) × 100. For a DKA duration of 5 h, the recommended monitoring frequency is five readings and 100% adherence. If the number of tasks is recorded as 3 or 7 for the same patient, the adherence would be derived as 60% or 140%, respectively.

#### 2.3.3 Complications during DKA treatment

The proportion of hypoglycaemia (blood glucose <4 mmol/L) during DKA was calculated using the following formula: (total number of hypoglycaemia/ total number of DKA) in the defined time. The proportions were calculated using the formula: total number of episodes for each complication/ total number of DKA in the defined time. Hypokalaemia, normokalaemia, and hyperkalaemia were <3.5, 3.5 to 5.5 and >5.5 mmol/L, respectively.

DKA duration was calculated as the time difference between DKA diagnosis and resolution and expressed in hours.

Length of stay was calculated as the time difference between admission and discharge expressed in days.

### 2.4 Analysis

Data were analysed using SPSS 28.0. Adherence to fluids, FRIII, glucose and ketone monitoring were calculated each quarter for each hospital. We then identified the proportion of patients within +/−20% (80%–120% adherence) of the JBDS‐IP recommendation. We calculated the proportion of hypoglycaemia, hypokalaemia, and hyperkalaemia each quarter. The median and IQRs for DKA duration in hours and length of stay in days were also calculated for these periods. We analysed the difference in all these parameters between the first (January–March 2020 for hospitals A, B, C, and D; January–March 2021 for hospitals E, F; and January–March 2022 for hospitals G, H, I, J and K) and the last quarter of QIP in this study (October–December 2022) using chi‐square test.

### 2.5 Ethical considerations

The QIP was registered with the Department of information governance in respective hospitals (Approval numbers: University Hospitals Birmingham [encompasses Queen Elizabeth Hospital Birmingham, Birmingham Heartlands Hospital and Good Hope Hospital]‐Clinical Audit Registration and Management Number‐12074, Sandwell and West Birmingham NHS Trust [encompasses Birmingham City Hospital and Sandwell Hospital]‐1538, Walsall Hospital‐ QI20‐21/LTC/01, Hereford County hospital‐QIP 010, New Cross hospital 16,468, Russel Hall hospital‐ Diab/QI/2022–23/08, Wirral University Teaching hospitals 6118 and Norfolk and Norwich University Hospitals‐DIAB‐22‐23‐A08).

## 3 RESULTS

We identified 1977 DKA episodes across 11 hospitals between January 2020 and December 2022. Hospitals are coded as A (*n* = 516), B (*n* = 218), C (*n* = 142), D (*n* = 163), E (*n* = 133), F (*n* = 226), G (*n* = 33), H (*n* = 106), I (*n* = 187), J (*n* = 122) and K (*n* = 133) to ensure anonymity and avoiding comparison. The cohort's median age was 45.0 years (IQR): (29.0–61.0 years). The ratio of women to men was 1: 1.29 (863 women and 1114 men).

### 3.1 Adherence of management to the national guideline

#### 3.1.1 FRIII

**FIGURE 2:**
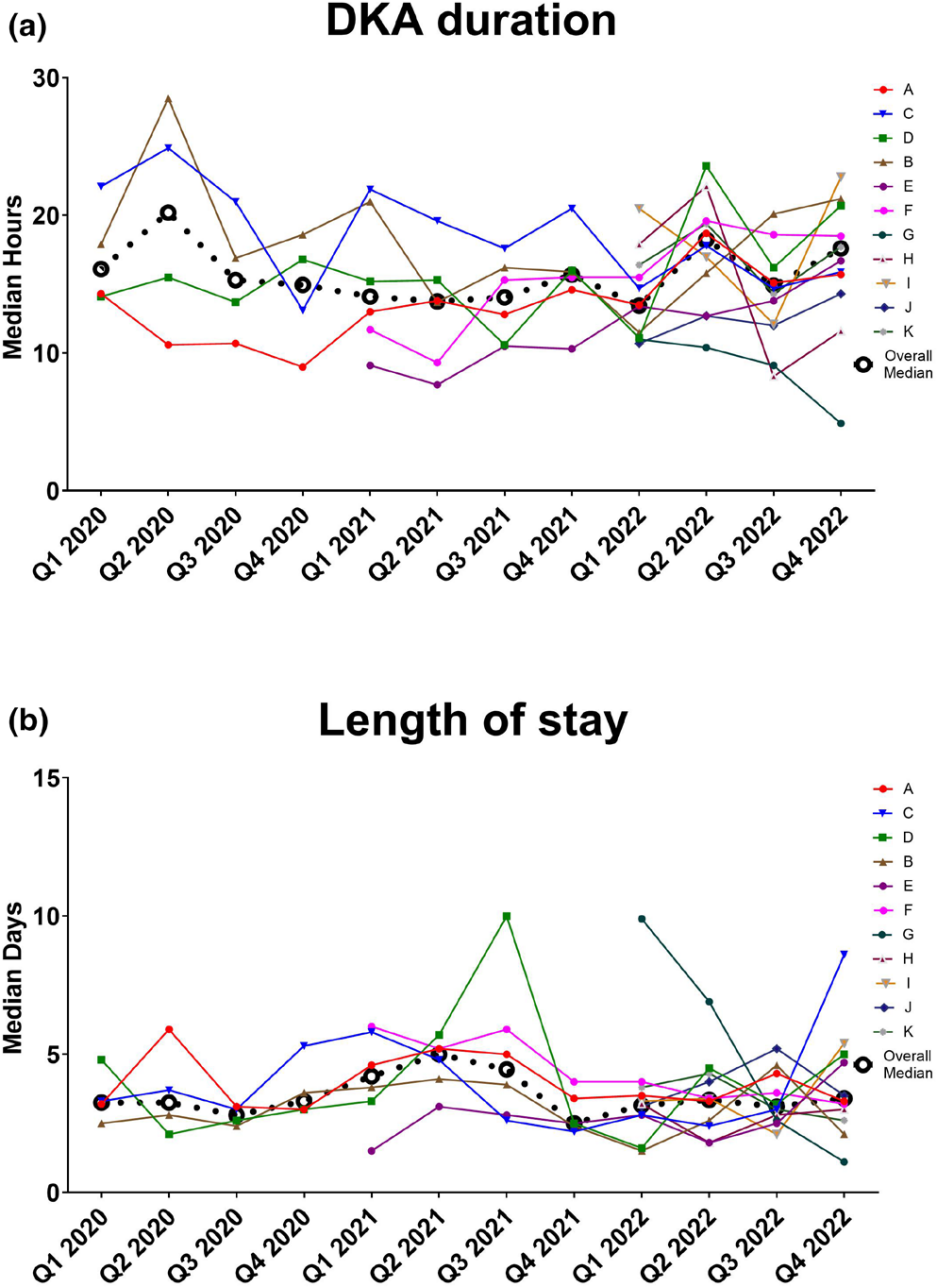
Trends in DKA outcome metrics across 11 Centres participating in the DEKODE initiative from January 2020 to December 2022: (a) Duration of DKA and (b) Length of Hospital Stay.

Hospitals C and G had more episodes prescribed FRIII within 80–120% of national recommendation at the end of QIP compared to the start of QIP (Start of QIP vs. End of QIP, p‐value; C‐ 88% vs. 100% *p* = <0.001; G‐ 91% vs. 100%, *p* = 0.013). However, hospitals B and D witnessed a decline in the number of people prescribed FRIII within 80–120% of recommendation (B‐ 100% vs. 50%, *p* < 0.001 D‐ 80% vs. 50%, p = <0.001) (Figure 2a and Table S2). The median adherence of FRIII in various hospitals during the QIP is described in Table S3.

#### 3.1.2 Fluids

Hospitals C, D, E, F, and G had more number of people administered fluids within 80–120% of national recommendation by the last quarter of the QIP (C‐ 23% vs. 75% *p* = <0.001; D‐ 27% vs. 60%, *p* = <0.001; E‐ 17 vs. 79% *p* = <0.001; F‐ 16% vs. 57%, *p* = <0.001; G‐ 36% vs. 75% *p* = <0.001) In hospital A, there was an effect towards improvement in the number of people administered fluids within 80%–120% of national recommendation (A‐ 39% vs. 48% *p* = 0.13) (Figure 2b and Table S4). The median adherence of fluids in various hospitals during the QIP is described in Table S5.

#### 3.1.3 Hourly glucose monitoring

Hospitals B, D, and G had notable increases in the number of people having hourly glucose monitoring (B‐ 11 vs. 38% *p* = <0.001; D‐ 36% vs. 56%, *p* = 0.05; G‐ 22% vs. 67% *p* = <0.001). A trend towards increased monitoring was seen in hospitals A, C and E (A‐ 40 vs. 44% p = <0.566; C‐ 17% vs. 25% *p* = 0.164; E‐ 67% vs. 70%, *p* = 0.647), but statistical significance was not reached (Table S6). The median adherence to hourly glucose monitoring in various hospitals during the QIP is described in Table S7.

#### 3.1.4 Hourly ketones monitoring

Hospital E witnessed an increase in the number of people having 80%–120% adherence in hourly ketone monitoring during the QIP (E‐ 55% vs. 73%, *p* = 0.008) whilst hospital D (70% vs. 19%, *p* = <0.00001) had the lowest number of DKA episodes within 80%–120% adherence in hourly ketone monitoring in the last quarter of the QIP compared to the first quarter (Table S8). The median commitment to hourly ketone monitoring in various hospitals during the QIP is described in Table S9.

### 3.2 Complications associated with DKA management

The proportion of hypoglycaemia: there were no significant differences in the ratio of DKA episodes having hypoglycaemia between the first and the last quarter of the QIP across all hospitals (Table S10).

#### 3.2.1 The proportion of hypokalaemia

Hospital A had a significantly higher number of hypokalaemia episodes between the first and the last quarter of the QIP (20.0% vs. 41.0%, *p* = 0.029). The detailed hospital results are in Table S11.

#### 3.2.2 The proportion of hyperkalaemia

Hospital C significantly increased the number of hyperkalaemia episodes between the first and the last quarter of the QIP (46.2% vs. 50.0%, *p* = 0.011). The detailed hospital results are in Table S12.

### 3.3 Trends of DKA outcomes

#### 3.3.1 DKA duration (hours)

Hospitals A, B, C, D, H, and I have significant variations in DKA duration across the QIP. Except for C and D, no significant differences in DKA duration were noticed in hospitals A‐K between the first and last quarter of QIP. (January–March 2020 vs. October–December 2022, p‐value: A‐ 14.3 vs. 15.7, p‐0.019; B‐ 17.9 vs. 21.2, p‐0.761; E‐ 22.1 vs. 51.9, p‐0.175; F‐14.1 vs. 20.7, p‐0.179; G‐ 11 vs. 4.9, p‐0.075, H‐ 17.9 vs. 11.6, p‐0.482; I‐ 20.5 vs. 22.8, p‐0.286; J‐ 10.7 vs. 14.3, p‐0.132, K‐ 16.4 vs. 17.7, p‐0.158). Hospitals C and D witnessed an increase in DKA duration by the end of QIP compared to their start in 2021. (C‐9.10 vs. 16.7, *p* < 0.001; D‐ 11.7 vs. 18.5, p < 0.001). Trends in DKA duration are depicted in Table S13.

#### 3.3.2 Length of stay (days)

Hospitals A, C and I showed significant variations in the length of stay across the QIP. Similar to DKA duration, hospitals C and D had an increased hospital stay by the end of QIP. (C‐ 4.8 vs. 5, p‐0.010; D‐1.5 vs. 2.1, p‐0.034). No significant change in the length of stay was observed across other hospitals between the start and the end of the QIP (A‐ 3.2 vs. 3.3, p‐0.466; B‐ 2.5 vs. 4.7, p‐0.136; E‐ 3.3 vs. 8.6, p‐0.056; F‐ 4.8 vs. 5.0, p‐0.159; G‐ 9.9 vs. 1.1, p‐0.080, H‐ 3.2 vs. 3.0, p‐0.968; I‐ 3.3 vs. 5.4, p‐0.464; J‐ 3.1 vs. 3.5, p‐0.854; K‐ 3.8 vs. 2.6, p‐0.115). Table S14 describes trends in length of stay.

## 4 DISCUSSION

We describe a QI model with capabilities to continuously monitor DKA care, thus facilitating early identification of changes in trends. We noticed a reduced variation in the quality of care for people with DKA in the DEKODE model. This is confirmed by increasing proportions of DKA episodes during which JBDS‐IP recommendations are adhered to. Despite improvements in adherence to JBDS‐IP recommendations, we did not notice a significant improvement in the median time to DKA resolution or length of stay across participating hospitals by the end of the reported QIP timeframe.

This could be attributed to multiple factors, medical and non‐medical. Fareen Ata et al. said that male‐gender, new‐onset DM, higher Charlson Comorbidity Index (CCI), lower haemoglobin, sodium and potassium, higher urea, longer DKA duration and intensive care admission could predict longer hospitalisation.^23^ In addition, implementing an intervention is a complex process which could be hindered by various individual, organisational, and system‐level barriers.^24^, ^25^ This highlights the need for future work on understanding the facilitators and barriers of improving DKA care beyond improving adherence to guidelines. The National Diabetes Inpatient Audit Report 2019 underscores a concerning trend: rising medication errors, insulin errors, and hospital acquired DKA.^26^ This growing body of evidence highlights the pressing need for sustained efforts to enhance the quality of care. Drawing insights from our study and other multisite quality improvement initiatives, like Project ECHO,^27^ can serve as valuable examples in addressing these challenges, especially in underserved communities.

Standardised systems of QIP resulted in reduced duplication of administrative work for audit registration, and a central analysis reduced the additional burden on participating hospitals so they could focus on implementing interventions to improve DKA care. These are important as initiating and maintaining QIP requires significant time and affects clinical work at the expense of patient care.^16^ Through standardised data collection and analysis with DEKODE, we created trends on key performance indicators and outcomes of DKA management. With more hospitals joining DEKODE, the data can be utilised more to consolidate evidence and inform future guideline amendments if required. By employing both quantitative and qualitative methods, including workload assessments through surveys, time tracking, and feedback mechanisms from healthcare professionals and administrative staff involved in DKA management through DEKODE study, future studies may comprehensively gauge any changes in workload, stress levels, and job satisfaction post‐implementation that may help in understanding the quality of life changes and will be pivotal for refining interventions, optimizing patient care, and sustaining the effectiveness of our QIPs through sustainable workflow and clear data collection processes.

In DEKODE, members of the participating hospitals meet virtually once a month to update on their local progress, discuss potential hindrances, share inputs, and agree on mutual deadlines on data submissions into the centralised DEKODE platform to facilitate timely analysis and feedback. The meetings served as a platform for exchanging ideas, support in planning interventions and assistance in interpreting feedback. The key to sustainability was attributed to the coordinated efforts by junior members through advanced planning and anticipation that resulted in improved handover to their successors.

Our QIP has some limitations. We did not measure the impact of individual interventions, hence being unable to determine their effects. We attempted coordinated efforts in delivering interventions simultaneously across hospitals to aid precise monitoring of improvements. However, this was not feasible given the varying availabilities and time constraints of junior doctor leads involved in the project. A systematic review on the effect of audit and feedback by Ivers et al. describe that interventions and feedback are likely to be effective when provided from a source that is a ‘supervisor or senior colleague’, and delivered at least ‘monthly’, in both a ‘verbal and written’ format, aiming to decrease rather than increase provider behaviours, and offers instructions with ‘both explicit goals and a specific action plan’.^13^ In DEKODE we deliver multifaceted interventions and regular feedback through emails and in‐person presentations every 3 months however the part of goals and action plans are left to the discretion of independent stakeholders based on their interpretation of trends at their respective hospitals. Peters et al. report that pre‐identifying barriers and utilising theories or frameworks in implementation planning can help improve outcomes.^24^ Henceforward, we aim to address this through mutual agreement between us and the individual stakeholders involved in DKA management. Our initiative, showcased through DEKODE, allows hospitals and healthcare providers to collaboratively learn and improve adherence to guidelines, fostering a culture of continuous improvement in patient care. Further studies should investigate additional medical and non‐medical factors, including characteristics of health professionals and patients, to study their impact on the outcomes. This approach could reveal whether certain groups might benefit more or require additional focus, thus elucidating the clinical implications.

This study utilised data from the DEKODE registry, a comprehensive database created to facilitate research into various diabetes‐related emergencies. Although this registry has been used in prior analyses to address different research aims (Ref: DME‐2024‐xxxx), the current study specifically examines how a cloud‐based performance feedback model improved DKA care across multiple hospitals in the UK. This approach follows standard epidemiological and registry‐based research practices, where large databases are employed for multiple investigations to enhance their utility and uncover new insights. To maintain methodological rigour and avoid redundancy, our study followed predefined objectives and employed robust statistical methods suited to the specific hypotheses being tested.

## 5 CONCLUSION

We describe a sustainable QI model that facilitates reliable monitoring of DKA trends across multiple hospitals through centralised data collection and analysis. Regular feedback on key performance indicators has shown potential in improving adherence to certain aspects of national guidelines, though not uniformly across all indicators of care. Future work is necessary to identify factors and strategies that influence or predict the adoption of evidence‐based interventions, providing regarding the impact of guidelines or recommendations.

## AUTHOR CONTRIBUTIONS

L.N.R and C.C are the joint‐first authors, contributing to all study aspects. PK conceptualised and supervised the delivery of all study aspects and critically reviewed the manuscript. All authors contributed substantially to drafting and approving the final draft of the manuscript. All the authors have reviewed and approved the final version. PK is the guarantor of this work and, as such, has full access to all the data in the study and takes responsibility for the integrity of the data and the accuracy of the data analysis. All members of DEKODE and DEVI Group contributed sufficiently to warrant authorship for this article.

## FUNDING INFORMATION

This research received no specific grant from any funding agency in the public, commercial or not‐for‐profit sectors. PK receives support from the National Institute for Health and Care Research (NIHR) through his Advanced Clinician Scientist Fellowship. Additionally, he is supported by the Midlands Patient Safety Research Collaboration (PSRC), and the NIHR‐supported Race, Equity, and Diversity in Careers Incubator. The views expressed in this study are those of the authors and do not necessarily reflect the official positions of the NIHR or the Department of Health and Social Care.

## CONFLICT OF INTEREST STATEMENT

The authors have nothing to report.

6

## Supporting information


Data S1.

## Data Availability

The data that support the findings of this study are available from the corresponding author upon reasonable request.
