# Supplementary material for: DEKODE—A cloud‐based performance feedback model improved DKA care across multiple hospitals in the UK
Source: Diabet Med. 2025 Feb 17;42(6):e70004. doi: 10.1111/dme.70004 (PMC12080979; doi:10.1111/dme.70004)
Supplement: Supplementary file 1 — Data S1. [file DME-42-e70004-s001.docx]

**Table S1: Table to explain the formulae used to determine the adherence to fluid prescription in line with DKA duration.**

| DKA duration in hours | Formula to determine the adherence to fluids prescription |
| --- | --- |
| 0-1 | Total volume of fluid in ml/1000ml)x100 |
| 1-3 | Total volume of fluid in ml/2000ml)x100 |
| 3-5 | (Total volume of fluid in ml/3000ml)x100 |
| 5-9 | Total volume of fluid in ml/4000ml)x100 |
| 9-13 | (Total volume of fluid in ml/5000ml)x100 |
| 13-19 | Total volume of fluid in ml/6000ml)x100 |
| 19-25 | Total volume of fluid in ml/7000ml)x100 |
| 25-31 | Total volume of fluid in ml/8000ml)x100 |
| for every 6 hours after | the denominator in the above formulae increases by 1000 |

**Table S2: Proportion of DKA episodes between 80% and 120% of the Joint British Diabetes Society- Inpatient recommendation for fixed rate intravenous insulin infusion prescription. Values expressed per cent.**

| **Timelines** | **A** | **B** | **C** | **D** | **E** | **F** | **G** | **H** | **I** | **J** | **K** | **Average** |
| --- | --- | --- | --- | --- | --- | --- | --- | --- | --- | --- | --- | --- |
| **Q1 2020** | 79.2 | 100.0 | 87.5 | 80.0 |  |  |  |  |  |  |  | 86.7 |
| **Q2 2020** | 81.1 | 77.8 | 93.3 | 80.0 |  |  |  |  |  |  |  | 83.0 |
| **Q3 2020** | 91.4 | 65.0 | 80.0 | 76.9 |  |  |  |  |  |  |  | 78.3 |
| **Q4 2020** | 91.7 | 72.7 | 55.6 | 83.3 |  |  |  |  |  |  |  | 76.0 |
| **Q1 2021** | 86.3 | 73.7 | 80.0 | 88.9 | 100.0 | 88.19 |  |  |  |  |  | 86.3 |
| **Q2 2021** | 90.8 | 84.0 | 71.4 | 45.5 | 26.7 | 87.5 |  |  |  |  |  | 67.7 |
| **Q3 2021** | 81.5 | 88.0 | 69.2 | 50.0 | 100.0 | 88.9 |  |  |  |  |  | 79.5 |
| **Q4 2021** | 54.8 | 69.0 | 90.0 | 78.6 | 93.3 | 75.0 |  |  |  |  |  | 76.8 |
| **Q1 2022** | 91.9 | 83.3 | 75.0 | 66.7 | 92.3 | 82.6 | 90.9 | 75.0 | 44.4 | 84.1 | 40.0 | 84.2 |
| **Q2 2022** | 77.8 | 87.5 | 81.8 | 75.0 | 100.0 | 78.6 | 100.0 | 100.10 | 85.1 | 88.0 | 0 | 87.5 |
| **Q3 2022** | 88.5 | 88.9 | 50.0 | 100.0 | 66.7 | 87.0 | 87.5 | 75.0 | 58.8 | 67.0 | 100.00 | 66.7 |
| **Q4 2022** | 80.4 | 50.0 | 100.00 | 50.0 | 97.0 | 85.7 | 100.0 | 100.0 | 26.7 | 84.0 | 100.00 | 83.9 |

**Table S3: Quarterly proportion across timelines of fixed rate intravenous insulin infusion prescription in hospitals A-K.**

| **Timelines** | **A**  **Median (IQR); n** | **B**  **Median (IQR); n** | **C**  **Median (IQR); n** | **D**  **Median (IQR); n** | **E**  **Median (IQR); n** | **F**  **Median (IQR); n** | **G**  **Median (IQR); n** | **H**  **Median (IQR); n** | **I**  **Median (IQR); n** | **J**  **Median (IQR); n** | **K**  **Median (IQR); n** |
| --- | --- | --- | --- | --- | --- | --- | --- | --- | --- | --- | --- |
| **Q1 2020** | 99.1(91.0-101.1); 45 | 100.0(93.4-104.4); 25 | 96.2 (86.1-100.0); 13 | 94.3 (78.2-103.2); 18 |  |  |  |  |  |  |  |
| **Q2 2020** | 99.6 (89.4-104.3); 36 | 100.0(96.4-112.6);19 | 100.0 (100.0-107.7); 15 | 100.9(88.5-109.5); 16 |  |  |  |  |  |  |  |
| **Q3 2020** | 98.0 (91.2-100); 37 | 97.2 (70.8-100.0);21 | 100.0(89.5-104.6); 11 | 96.4 (80.9-100.6); 18 |  |  |  |  |  |  |  |
| **Q4 2020** | 102.6(97.6-109.2); 35 | 100.0(93.3-103.4); 11 | 100.0(82.5-120.5); 10 | 100.0(86.5-102.1); 20 |  |  |  |  |  |  |  |
| **Q1 2021** | 100.0 (92-111.1); 52 | 100.0(80.5–117.6); 21 | 96.9 (90.9-103.7); 11 | 100.0(95.2-108.9); 17 | 102.8 (100.0-102.9); 12 | 100.0(99.8-100.4); 21 |  |  |  |  |  |
| **Q2 2021** | 100.0(97.2-107.1); 55 | 96.8 (85.6-100); 26 | 95.8 (81.3-101.5); 14 | 100.0(89.2-144.2); 15 | 99.2 (94.6-120.5); 15 | 100.0(99.1-108.3); 17 |  |  |  |  |  |
| **Q3 2021** | 100.0(93.2-107.5); 27 | 98.4 (90.4-101.7); 26 | 100.0(92.6-123.8); 13 | 91.6 (79.8-101.3); 4 | 99.5 (95.1-100.0); 22 | 97.5 (92.9-102.4); 19 |  |  |  |  |  |
| **Q4 2021** | 98.5 (62.0-109.9); 41 | 97.2 (84.3-100.0); 17 | 93.9 (84.7-100.0); 11 | 99.9 (93.2-111.8); 8 | 100.0(95.3-100.0); 16 | 99.8 (85.8-100.8); 32 |  |  |  |  |  |
| **Q1 2022** | 101.5 (100.0-107.4); 36 | 101.0(74.6-110.9); 14 | 87.6 (1.5-103.1); 12 | 87.0 (41.6-.); 4 | 100.0(94.9-100.0); 12 | 100.0(96.4-105.9); 34 | 100.0 (84.8 - 115.9); 9 | 95.0 (87.8 - 106.6); 28 | 102.4 (99.3 - 106.8); 13 | 95.6 (90.2 - 103.8); 18 | 92.6 (61.3 - 114.5); 5 |
| **Q2 2022** | 99.4 (89.6 - 103.5); 37 | 101.3 (90.9 - 106.2); 7 | 91.4 (84.4 - 100.9); 10 | 101.1 (85.5 - 117.3); 4 | 100.0 (98.9 - 110.0); 9 | 100.0 (96.5 - 112.5); 28 | 100.0 (97.2 - 104.5); 7 | 100.0 (92.7 - 108.3); 5 | 97.0 (87.0 - 105.8); 23 | 97.6 (93.3 - 101.6); 15 | NA |
| **Q3 2022** | 103.0 (99.4 - 113.2); 22 | 100.0 (81.7 - 100.0); 9 | 70.5 (35.9 - 111.0); 4 | 98.7 (94.6 - 103.0); 7 | 100.0 (86.0 - 122.4); 7 | 100.0(100.0-111.7); 21 | 100.4(100.0 - N/A); 2 | 94.3 (88.2 - 104.6); 16 | 94.6 (81.4 - 125.0); 7 | 94.0 (76.8 - 103.5); 28 | 101.5 (100.0 - N/A); 2 |
| **Q4 2022** | 100.0 (99.3 - 101.6); 56 | 82.2 (76.9 - 96.9); 4 | 96.1 (84.8 - 101.1); 42 | 117.0(102.0 - 137.8); 6 | 100.0 (98.4 - 101.8); 33 | 100.0(97.9-103.2);42 | 96.8 (96.8 - 96.8); 1 | 98.0 (92.3 - 102.9); 16 | 96.6 (92.7 - 100.0); 15 | 97.8 (91.8 - 100.2); 52 | 102.3 (102.3 - 102.3); 1 |

**Table S4: Proportion of DKA episodes between 80% and 120% of the Joint British Diabetes Society- Inpatient recommendation for fluids prescription. Values expressed per cent.**

| **Timelines** | **A** | **B** | **C** | **D** | **E** | **F** | **G** | **H** | **I** | **J** | **K** | **Average** |
| --- | --- | --- | --- | --- | --- | --- | --- | --- | --- | --- | --- | --- |
| **Q1 2020** | 38.6 | 60.0 | 23.1 | 31.3 |  |  |  |  |  |  |  | 37.3 |
| **Q2 2020** | 21.6 | 31.6 | 73.3 | 14.3 |  |  |  |  |  |  |  | 39.5 |
| **Q3 2020** | 19.4 | 43.0 | 45.5 | 37.5 |  |  |  |  |  |  |  | 30.3 |
| **Q4 2020** | 41.7 | 54.5 | 40.0 | 33.3 |  |  |  |  |  |  |  | 43.8 |
| **Q1 2021** | 52.9 | 58.8 | 50.0 | 29.4 | 16.7 | 15.8 |  |  |  |  |  | 38.0 |
| **Q2 2021** | 52.7 | 32.0 | 42.9 | 22.2 | 33.3 | 29.4 |  |  |  |  |  | 36.5 |
| **Q3 2021** | 29.6 | 88.5 | 69.2 | 25.0 | 47.6 | 31.6 |  |  |  |  |  | 48.2 |
| **Q4 2021** | 36.6 | 41.2 | 36.4 | 25.0 | 62.5 | 18.8 |  |  |  |  |  | 36.8 |
| **Q1 2022** | 24.3 | 40.0 | 33.3 | 20.0 | 53.8 | 41.2 | 36.4 | 36.7 | 24.0 | 47.6 | 25.0 | 34.1 |
| **Q2 2022** | 55.0 | 9.1 | 33.3 | 75.0 | 44.4 | 51.4 | 42.9 | 50.0 | 32.8 | 43.8 | 9.0 | 43.7 |
| **Q3 2022** | 44.4 | 50.0 | 40.0 | 66.7 | 40.0 | 39.1 | 37.5 | 41.4 | 38.9 | 39.3 | 36.0 | 43.7 |
| **Q4 2022** | 48.2 | 50.0 | 75.0 | 60.0 | 78.8 | 57.1 | 75.0 | 52.0 | 25.0 | 61.4 | 30.0 | 58.2 |

**Table S5: Quarterly proportion across timelines of fluids prescription in hospitals A-K.**

| **Timelines** | **A**  **Median (IQR); n** | **B**  **Median (IQR); n** | **C**  **Median (IQR); n** | **D**  **Median (IQR); n** | **E**  **Median (IQR); n** | **F**  **Median (IQR); n** | **G**  **Median (IQR); n** | **H**  **Median (IQR); n** | **I**  **Median (IQR); n** | **J**  **Median (IQR); n** | **K**  **Median (IQR); n** |
| --- | --- | --- | --- | --- | --- | --- | --- | --- | --- | --- | --- |
| **Q1 2020** | 83.3 (68.3-100.0) ; 45 | 90.5 (55.0-133.3) ; 25 | 69.6 (54.7-112.5) ; 13 | 92.9 (55.0-122.5) ; 18 |  |  |  |  |  |  |  |
| **Q2 2020** | 78.9 (39.4-114.4) ;36 | 104.2 (67.3-150.0) ; 19 | 100.0 (87.5-141.7) ; 15 | 72.5 (54.2-112.5) ; 16 |  |  |  |  |  |  |  |
| **Q3 2020** | 100.0 (65.6-151.9) ;37 | 110.0 (79.2-138.1) ; 21 | 91.7 (52.1-114.3) ; 11 | 100.0 (76.3-120.0) ; 18 |  |  |  |  |  |  |  |
| **Q4 2020** | 101.7 (80.0-143.3) ; 35 | 83.3 (75.0-108.3) ; 11 | 101.3 (77.7-153.2) ; 10 | 100.0 (80.0-133.3) ; 20 |  |  |  |  |  |  |  |
| **Q1 2021** | 91.5 (70.5-109.8) ; 52 | 100.0 (88.3-117.1) ; 21 | 107.3 (91.5-122.2) ; 11 | 120.0 (89.2-150) ; 17 | 156.8 (109.6-178.4) ; 12 | 135.0 (122.0-145.0) ; 21 |  |  |  |  |  |
| **Q2 2021** | 86.4 (71.0-108.6) ; 55 | 100.0 (65.0-120) ; 26 | 96.9 (82.5-148.7) ; 14 | 150.0 (116.7-177.5) ; 15 | 126.3 (83.0-152.5) ; 15 | 66.7 (45.0-100.0) ; 17 |  |  |  |  |  |
| **Q3 2021** | 69.5 (41.3-91.3) ; 27 | 102.8 (93.8-125.7) ; 26 | 100.0 (79.2-112.1) ; 13 | 133.3 (81.3-201); 4 | 96.5 (78.1-103.5) ; 22 | 66.7 (57.1-109.3) ; 19 |  |  |  |  |  |
| **Q4 2021** | 76.7 (58.6-93.3) ; 41 | 100.0 (82.9-122.5) ; 17 | 90.0 (62.5-100.0) ; 11 | 120.8 (80.8-157.5) ; 8 | 102.1 (89.8-121.4) ; 16 | 81.0 (54.0-134.4) ; 32 |  |  |  |  |  |
| **Q1 2022** | 77.3 (64.1-111.9) ; 36 | 91.7 (68.6-140) ; 14 | 108.3 (80.8-125); 12 | 75.0(50.0-89.3); 4 | 101.7 (92.9-121.1) ; 12 | 94.2 (70.0-120.4) ; 34 | 54.1 (39.8 - 90.0); 8 | 90.0 (70.0 - 125.0); 39 | 93.3 (67.9 - 128.8); 28 | 83.0 (62.9 - 109.4): 20 | 64.6 (53.1 - 104.2); 4 |
| **Q2 2022** | 80.0 (57.8 - 89.3); 14 | 125.0 (62.5 - 150.0); 7 | 125.0 (94.7 - 150.0); 9 | N/A (N/A - N/A); 0 | 82.1 (72.6 - 98.4); 4 | 97.6 (77.8 - 110.9); 14 | 100.0 (78.0 - 135.6); 5 | 72.9 (50.0 - 111.1); 10 | 129.3 (100.0 - 165.0); 26 | 85.7 (66.7 - 116.7); 15 | 50.0 (40.0 - 68.3); 21 |
| **Q3 2022** | 71.0 (58.3 - 88.8); 23 | 85.0 (76.2 - 106.7); 10 | 83.3 (78.3 - 125.0); 5 | 108.3 (100.0 - 138.1); 8 | 79.3 (62.8 - 85.0); 7 | 75.8 (56.7 - 100.8); 21 | 109.5 (81.3 - N/A) | 125.0 (103.6 - 155.0); 29 | 130.0 (113.8 - 175.0); 17 | 80.0 (50.0 - 100.0); 29 | 66.7 (50.0 - 100.0); 19 |
| **Q4 2022** | 96.0 (66.6 - 128.5); 56 | 100.0 (67.2 - 200.7); 8 | 125.0 (120.0 - 212.5); 4 | 140.0 (106.3 - 175.0); 9 | 111.0 (92.3 - 122.0); 33 | 82.1(63.8-92.7); 42 | 40.0 (40.0 - 40.0); 1 | 90.0 (77.5 - 122.5); 25 | 121.8 (100.0 - 142.9); 36 | 100.0 (80.0 - 134.6); 53 | 75.0 (66.7 - 83.9); 26 |

**Table S6: Proportion of DKA episodes between 80% and 120% of the Joint British Diabetes Society- Inpatient recommendation for hourly glucose monitoring. Values expressed per cent.**

| **Timelines** | **A** | **B** | **C** | **D** | **E** | **F** | **G** | **H** | **I** | **J** | **K** | **Average** |
| --- | --- | --- | --- | --- | --- | --- | --- | --- | --- | --- | --- | --- |
| **Q1 2020** | 39.7 | 11.1 | 16.7 | 36.3 |  |  |  |  |  |  |  | 26.0 |
| **Q2 2020** | 37.8 | 33.3 | 26.7 | 32.8 |  |  |  |  |  |  |  | 32.8 |
| **Q3 2020** | 47.2 | 43.0 | 54.5 | 19.4 |  |  |  |  |  |  |  | 41.0 |
| **Q4 2020** | 27.3 | 63.6 | 50.0 | 43.3 |  |  |  |  |  |  |  | 46.0 |
| **Q1 2021** | 45.1 | 45.0 | 60.0 | 35.0 | 66.7 | 70.0 |  |  |  |  |  | 53.7 |
| **Q2 2021** | 51.9 | 40.0 | 61.5 | 26.8 | 46.7 | 58.8 |  |  |  |  |  | 47.8 |
| **Q3 2021** | 44.4 | 64.0 | 53.8 | 25.0 | 66.7 | 52.6 |  |  |  |  |  | 51.2 |
| **Q4 2021** | 36.6 | 56.3 | 63.6 | 52.7 | 60.0 | 24.0 |  |  |  |  |  | 49.0 |
| **Q1 2022** | 48.1 | 33.3 | 40.0 | 50.0 | 69.2 | 48.1 | 22.2 | 24.2 | 43.5 | 58.8 | 23.2 | 43.6 |
| **Q2 2022** | 50.0 | 33.3 | 61.5 | 42.9 | 62.5 | 70.6 | 33.3 | 60.0 | 33.3 | 57.1 | 25.0 | 50.5 |
| **Q3 2022** | 66.7 | 33.3 | 40.0 | 88.9 | 40.0 | 47.8 | 57.1 | 14.3 | 17.9 | 37.5 | 37.4 | 44.4 |
| **Q4 2022** | 44.0 | 37.5 | 25.0 | 55.6 | 69.7 | 61.9 | 66.7 | 33.3 | 37.1 | 30.8 | 32.3 | 46.3 |

**Table S7: Quarterly proportion across timelines of hourly glucose monitoring in hospitals A-K.**

| **Timelines** | **A**  **Median (IQR); n** | **B**  **Median (IQR); n** | **C**  **Median (IQR); n** | **D**  **Median (IQR); n** | **E**  **Median (IQR); n** | **F**  **Median (IQR); n** | **G**  **Median (IQR); n** | **H**  **Median (IQR); n** | **I**  **Median (IQR); n** | **J**  **Median (IQR); n** | **K**  **Median (IQR); n** |
| --- | --- | --- | --- | --- | --- | --- | --- | --- | --- | --- | --- |
| **Q1 2020** | 118.1(94.2-145.8) ; 45 | 56.7 (40.9-67.2) ; 25 | 55.8 (29.6-75.8) ; 13 | 127.0(99.6-169.5) ; 18 |  |  |  |  |  |  |  |
| **Q2 2020** | 102.2 (85.7-142.2) ; 36 | 80.0 (69.8-114.6) ; 19 | 76.5 (64.3-94.4) ; 15 | 91.3 (51.5-154.2) ; 16 |  |  |  |  |  |  |  |
| **Q3 2020** | 117.8 (98.9-134) ; 37 | 99.8 (67.3-113.8) ;21 | 85.6 (55.2-94.8) ; 11 | 130.1 (107.2-150.2) ; 18 |  |  |  |  |  |  |  |
| **Q4 2020** | 124.2 (90.6-152.2) ; 35 | 82.2 (59.0-96.9) ;11 | 88.7 (67.3-118.1) ; 10 | 113.5 (89.6-147.5) ; 20 |  |  |  |  |  |  |  |
| **Q1 2021** | 112.1 (90.1-129.5) ; 52 | 89.7 (63.5-119.1) ; 21 | 97.1 (83.5-108.1) ; 11 | 112.1 (75.8-145.9) ; 17 | 101.4 (93.2-118.0) ; 12 | 94.6 (79.6-108.7) ; 21 |  |  |  |  |  |
| **Q2 2021** | 113.7 (98.5-144.9) ; 55 | 82.2 (66.9-98.6) ; 26 | 84.5 (62.9-104.6) ; 14 | 142.0 (91.3-15.3) ; 15 | 101.9 (55.6-121.0) ; 15 | 87.0 (74.2-98.1) ; 17 |  |  |  |  |  |
| **Q3 2021** | 110.9 (91.5-146.9) ; 27 | 90.0 (78.7-104.7) ; 26 | 102.6 (80.0-120.6) ; 13 | 168.9 (101.1-221.2) ; 4 | 92.6(81.3-113) ; 16 | 95.8 (71.2-104.2) ; 19 |  |  |  |  |  |
| **Q4 2021** | 115.1 (88.5-151.4) ; 41 | 95.4 (68.4-100.0) ; 17 | 91.8(68.3-99.9) ; 11 | 110.5 (89.9-136.4) ; 8 | 84.7 (78.6-95.7) ; 22 | 73.1 (53.6-115.3) ; 32 |  |  |  |  |  |
| **Q1 2022** | 106.0 (77.6-135.6) ; 36 | 123.0 (101.9-104.8) ; 14 | 109.5 (86.6-138.2) ; 12 | 84.5 (56.8-218.7) ; 4 | 84.0 (71.8-96) ; 12 | 99.2 (70.3-135.2) ; 34 | 60.9 (57.4 - 108.2); 8 | 53.6 (36.2 - 86.4); 33 | 77.3 (46.0 - 114.1); 21 | 98.9 (92.7 - 120.0); 16 | 64.3 (45.1 - 91.8); 34 |
| **Q2 2022** | 105.4 (82.6 - 130.7); 38 | 118.7 (64.7 - 154.6); 10 | 103.4 (80.3 - 116.8); 14 | 77.9 (46.5 - 104.6); 7 | 95.3 (76.8 - 111.3); 8 | 91.2 (74.7 - 103.3); 35 | 92.4 (63.6 - 123.3); 7 | 82.0 (57.8 - 82.8); 5 | 89.0 (61.2 - 116.1); 19 | 104.0 (83.3 - 120.5); 13 | 64.9 (54.6 - 81.0) 31 |
| **Q3 2022** | 95.2 (81.0 - 112.3); 23 | 101.4 (62.9 - 135.2); 10 | 94.2 (60.8 - 120.4); 5 | 107.9 (95.5 - 112.9); 8 | 87.3 (68.5 - 156.9); 7 | 86.2 (69.1 - 108.3); 21 | 86.9 (68.2 - N/A); 2 | 157.0 (96.3 - 280.5); 28 | 72.3 (49.5 - 136.9); 8 | 81.1 (74.1 - 100.1); 25 | 76.0 (56.6 - 100.5); 24 |
| **Q4 2022** | 99.9 (83.3 - 125.3); 50 | 96.6 (62.6 - 137.0); 8 | 52.1 (31.7 - 81.9); 4 | 104.7 (84.5 - 117.4); 9 | 92.7 (83.8 - 102.1); 33 | 88.8(61.6-98.4); 42 | 103.1 (103.1 - 103.1); 1 | 114.5 (86.4 - 178.1); 24 | 78.7 (53.5-104.9);35 | 104.3 (75.2 - 122.3); 49 | 66.0 (44.8 - 92.5); 28 |

**Table S8: Proportion of DKA episodes between 80% and 120% of the Joint British Diabetes Society- Inpatient recommendation for hourly ketone monitoring. Values expressed per cent.**

| **Timelines** | **A** | **B** | **C** | **D** | **E** | **F** | **G** | **H** | **I** | **J** | **K** | **Average** |
| --- | --- | --- | --- | --- | --- | --- | --- | --- | --- | --- | --- | --- |
| **Q1 2020** | 9.0 | 22.2 | 0 | 38.5 |  |  |  |  |  |  |  | 17.3 |
| **Q2 2020** | 31.4 | 17.6 | 6.7 | 54.5 |  |  |  |  |  |  |  | 27.8 |
| **Q3 2020** | 27.8 | 24.0 | 9.1 | 62.5 |  |  |  |  |  |  |  | 31.0 |
| **Q4 2020** | 27.8 | 0 | 10.0 | 64.3 |  |  |  |  |  |  |  | 25.5 |
| **Q1 2021** | 27.5 | 35.0 | 10.0 | 29.4 | 54.5 | 70.0 |  |  |  |  |  | 37.7 |
| **Q2 2021** | 14.8 | 28.0 | 30.8 | 55.6 | 30.8 | 58.8 |  |  |  |  |  | 36.7 |
| **Q3 2021** | 26.9 | 24.0 | 30.8 | 0 | 66.7 | 36.8 |  |  |  |  |  | 31.0 |
| **Q4 2021** | 17.5 | 5.9 | 9.1 | 33.3 | 73.3 | 24.0 |  |  |  |  |  | 27.7 |
| **Q1 2022** | 25.7 | 42.9 | 33.3 | 20.0 | 69.2 | 33.3 | 27.3 | 8.6 | 47.1 | 55.0 | 0 | 36.2 |
| **Q2 2022** | 8.1 | 27.3 | 20.0 | 42.9 | 63.5 | 20.6 | 28.6 | 25.0 | 30.8 | 37.5 | 0 | 30.5 |
| **Q3 2022** | 8.0 | 50.0 | 0 | 55.6 | 33.3 | 31.8 | 0 | 17.9 | 41.2 | 29.6 | 100.0 | 26.8 |
| **Q4 2022** | 23.2 | 25.0 | 0 | 44.4 | 72.7 | 19.0 | 33.3 | 45.9 | 40.0 | 18.2 | 0 | 32.0 |

**Table S9: Quarterly proportion across timelines of hourly ketone monitoring in hospitals A-K.**

| **Timelines** | **A**  **Median (IQR); n** | **B**  **Median (IQR); n** | **C**  **Median (IQR); n** | **D**  **Median (IQR); n** | **E**  **Median (IQR); n** | **F**  **Median (IQR); n** | **G**  **Median (IQR); n** | **H**  **Median (IQR); n** | **I**  **Median (IQR); n** | **J**  **Median (IQR); n** | **K**  **Median (IQR); n** |
| --- | --- | --- | --- | --- | --- | --- | --- | --- | --- | --- | --- |
| **Q1 2020** | 62.7 (42.0-72.9) ; 45 | 7.5 (4.2-13.2) ; 25 | 12.6 (9.4-21.8) ; 13 | 13.8 (10.9-19.2) ; 18 |  |  |  |  |  |  |  |
| **Q2 2020** | 62.3 (25.6-86.9) ; 36 | 12.6 (9.3-19.6) ; 19 | 15.0 (9.3-20.6) ; 15 | 12.6 (5.5-30.5) ; 16 |  |  |  |  |  |  |  |
| **Q3 2020** | 63.8 (42.9-83.2) ; 37 | 10.3 (7.0-15.0) ; 21 | 9.7 (6.8-12.7) ; 11 | 14.8 (9.0-19.7) ;18 |  |  |  |  |  |  |  |
| **Q4 2020** | 5.8 (4.1-8.1) ; 35 | 9 (5.8-15.0) ; 11 | 7.6 (4.3-11.0) ; 10 | 14.8 (9.2-16.6) ; 20 |  |  |  |  |  |  |  |
| **Q1 2021** | 60.5 (39.3-81.7) ; 52 | 68.8 (50.4-88.0) ; 21 | 66.6 (53.5-81) ; 11 | 14.6 (11.0-27.0) ; 17 | 100.8 (92.3-120.7) ; 12 | 92.5 (79.6-106.5) ; 21 |  |  |  |  |  |
| **Q2 2021** | 62.8 (45.6-75.3) ; 55 | 69.0 (50.1-85.9) ; 26 | 67.6 (42.2-85.8) ; 14 | 99.4 (48.3-113.7) ; 15 | 81.4 (54.6-119.5) ; 15 | 87.0 (74.2-98.1) ; 17 |  |  |  |  |  |
| **Q3 2021** | 61.4 (44.4-84.5) ; 27 | 67.0 (55.5-85.7) ; 26 | 79.3 (55.7-89.1) ; 13 | 135.6 (77.6-151.1) ; 4 | 90.1 (79.7-107.8) ; 22 | 96.0 (64.6-105.9) ; 19 |  |  |  |  |  |
| **Q4 2021** | 59.3 (47.0-76.8) ; 41 | 60.4 (54.9-74.3) ; 17 | 52.2(43.5-70.5) ; 11 | 86.2 (66.8-115.5) ; 8 | 84.7 (78.6-98.0) ; 16 | 58.8 (35.9-88.3) ; 32 |  |  |  |  |  |
| **Q1 2022** | 59.0 (43.1-82.7) ; 26 | 104.8 (79.4-126.6) ; 14 | 71.1 (63.8-87.7) ; 12 | 76.5 (47.3-218.7) ; 4 | 84.0 (71.8-96.0) ; 12 | 73.7 (58.1-93.7) ; 34 | 58.1 (50.4 - 98.4); 8 | 39.8 (27.8 - 76.1); 32 | 44.5 (25.2 - 84.6); 27 | 92.5 (72.0 - 101.1); 19 | 27.7 (27.7 - 27.7); 1 |
| **Q2 2022** | 59.0 (47.2 - 73.7); 38 | 68.0 (44.9 - 93.4); 10 | 60.0 (38.4 - 84.3); 14 | 77.9 (60.9 - 104.6); 7 | 95.3 (76.8 - 111.3); 8 | 67.6 (56.2 - 79.9); 35 | 59.4 (45.5 - 95.9); 7 | 49.3 (24.9 - 82.1); 8 | 55.6 (27.6 - 84.4); 25 | 79.0 (73.7 - 87.9); 15 | NA |
| **Q3 2022** | 60.2 (46.2 - 72.6); 23 | 71.4 (49.8 - 85.1); 10 | 63.7 (37.4 - 101.2); 5 | 86.6 (64.6 - 93.5); 8 | 75.9 (54.7 - 91.5); 7 | 75.6 (55.8 - 86.0); 21 | 32.9 (17.0 - N/A); 2 | 72.8 (55.3 - 134.1); 28 | 82.7 (45.6 - 102.2); 14 | 70.0 (56.6 - 89.4); 28 | 87.5 (87.5 - 87.5); 27 |
| **Q4 2022** | 63.6 (43.3 - 83.1); 56 | 79.5 (45.5 - 121.9); 8 | 22.3 (20.3 - 81.9); 4 | 89.6 (57.7 - 98.0); 9 | 84.9 (75.7 - 90.8); 33 | 68.1(60.3-79.0);42 | 61.9 (61.9 - 61.9); 1 | 76.4 (59.5 - 96.0); 22 | 39.7 (29.1 - 66.5); 35 | 70.5 (55.4 - 94.0); 52 | NA |

**Table S10: Proportion of admissions where hypoglycaemia occurred during DKA management in hospitals A-K across timelines. n- number of DKA episodes.**

| **Timelines** | **A %(n)** | **B %(n)** | **C %(n)** | **D %(n)** | **E %(n)** | **F %(n)** | **G %(n)** | **H %(n)** | **I %(n)** | **J %(n)** | **K %(n)** | **Average(%)** |
| --- | --- | --- | --- | --- | --- | --- | --- | --- | --- | --- | --- | --- |
| **Q1 2020** | 9.1(45) | 17.9 (25) | 23.5 (13) | 35 (18) |  |  |  |  |  |  |  | 18.3 |
| **Q2 2020** | 8.1 (36) | 30.0 (19) | 23.5 (15) | 18.8 (16) |  |  |  |  |  |  |  | 17.8 |
| **Q3 2020** | 16.7 (37) | 16.0 (21) | 15.4 (11) | 16.7 (18) |  |  |  |  |  |  |  | 16.3 |
| **Q4 2020** | 56.5 (35) | 25.0 (11) | 10.0 (10) | 10.0 (20) |  |  |  |  |  |  |  | 36.4 |
| **Q1 2021** | 9.6 (52) | 19.0 (21) | 9.1 (11) | 16.7 (17) | 16.7 (12) | 9.5 (21) |  |  |  |  |  | 12.5 |
| **Q2 2021** | 7.3 (55) | 15.4 (26) | 21.4(14) | 6.7 (15) | 6.7 (15) | 0 (17) |  |  |  |  |  | 9.1 |
| **Q3 2021** | 13.5 (27) | 7.7 (26) | 7.7 (13) | 40.0 (4) | 13.6 (22) | 30.0 (19) |  |  |  |  |  | 15.4 |
| **Q4 2021** | 4.4 (41) | 29.4 (17) | 45.5 (11) | 27.8 (8) | 17.6 (16) | 18.8 (32) |  |  |  |  |  | 18.6 |
| **Q1 2022** | 2.7 (36) | 7.1 (14) | 25.0 (12) | 0 (4) | 15.4 (12) | 20.6 (34) | 0 (9) | 7.5 (41) | 30.2 (28) | 4.8 (20) | 32.4 (34) | 16.6 |
| **Q2 2022** | 12.5 (41) | 0 (10) | 0 (14) | 12.5 (7) | 11.1 (9) | 17.1 (35) | 0 (7) | 22.2 (10) | 24.1 (26) | 12.5 (15) | 26.5 (33) | 16.5 |
| **Q3 2022** | 14.8 (23) | 20.0 (10) | 40.0 (5) | 11.1 (8) | 10.0 (7) | 0 (21) | 11.0 (2) | 13.8 (29) | 25.0 (16) | 17.9 (29) | 6.9 (27) | 14.6 |
| **Q4 2022** | 26.7 (56) | 62.5 (8) | 25.0 (4) | 9.1 (9) | 8.6 (33) | 14.0 (25) | 0 (2) | 16.0 (25) | 25.0 (36) | 17.5 (53) | 8.8 (32) | 18.3 |

**Table S11: Proportion of admissions where hypokalaemia occurred during DKA management in hospitals A-K across timelines. n- number of DKA episodes.**

| **Timelines** | **A %(n)** | **B %(n)** | **C %(n)** | **D %(n)** | **E %(n)** | **F %(n)** | **G %(n)** | **H %(n)** | **I %(n)** | **J %(n)** | **K %(n)** | **Average(%)** |
| --- | --- | --- | --- | --- | --- | --- | --- | --- | --- | --- | --- | --- |
| **Q1 2020** | 20.5 (45) | 21.4 (25) | 35.3 (13) | 45.0 (18) |  |  |  |  |  |  |  | 27.5 |
| **Q2 2020** | 40.5 (36) | 40.0 (19) | 35.3 (15) | 31.3 (16) |  |  |  |  |  |  |  | 37.8 |
| **Q3 2020** | 33.3 (37) | 36.0 (21) | 69.2 (11) | 27.8 (18) |  |  |  |  |  |  |  | 38.0 |
| **Q4 2020** | 10.9 (35) | 41.7 (11) | 30 (10) | 55.0 (20) |  |  |  |  |  |  |  | 27.3 |
| **Q1 2021** | 25.0 (52) | 47.6 (21) | 45.5 (11) | 38.9 (17) | 58.3(12) | 38.1 (21) |  |  |  |  |  | 36.8 |
| **Q2 2021** | 20.0 (55) | 26.9 (26) | 42.9 (14) | 20.0 (15) | 33.3 (15) | 33.3 (17) |  |  |  |  |  | 26.6 |
| **Q3 2021** | 48.6 (27) | 34.6 (26) | 38.5 (13) | 80.0 (4) | 36.4 (22) | 55.0 (19) |  |  |  |  |  | 44.7 |
| **Q4 2021** | 20.7(41) | 29.4 (17) | 63.6 (11) | 55.6 (8) | 23.5 (16) | 43.8 (32) |  |  |  |  |  | 37.1 |
| **Q1 2022** | 18.9 (36) | 35.7 (14) | 33.3 (12) | 40.0 (4) | 15.4 (12) | 38.2 (34) | 27.3 (9) | 27.5 (36) | 47.2(28) | 52.4 (20) | 24.3 (34) | 33.2 |
| **Q2 2022** | 30.0 (41) | 45.5 (10) | 60.0 (14) | 12.5 (7) | 11.1 (9) | 37.1 (35) | 28.6 (7) | 33.3 (10) | 32.8 (26) | 25.0 (15) | 32.4 (33) | 33.1 |
| **Q3 2022** | 25.9 (23) | 50.0 (10) | 0 (5) | 55.6 (8) | 40.0 (7) | 34.8 (21) | 22.2 (2) | 41.4 (29) | 45.0 (16) | 46.4 (28) | 27.6 (27) | 37.4 |
| **Q4 2022** | 40.0 (56) | 50.0 (8) | 50.0 (4) | 54.5 (9) | 37.1 (33) | 44.2 (19) | 0(2) | 44.0 (25) | 55.6 (36) | 22.8 (53) | 14.7 (32) | 36.9 |

**Table S12: Proportion of admissions where hyperkalaemia occurred during DKA management in hospitals A-K across timelines. n- number of DKA episodes.**

| **Timelines** | **A %(n)** | **B %(n)** | **C %(n)** | **D %(n)** | **E %(n)** | **F %(n)** | **G %(n)** | **H %(n)** | **I %(n)** | **J %(n)** | **K %(n)** | **Average(%)** |
| --- | --- | --- | --- | --- | --- | --- | --- | --- | --- | --- | --- | --- |
| **Q1 2020** | 22.7 (45) | 25.0 (25) | 11.8 (13) | 40.0 (18) |  |  |  |  |  |  |  | 24.8 |
| **Q2 2020** | 27.0 (36) | 30.0 (19) | 35.3 (15) | 18.8 (16) |  |  |  |  |  |  |  | 27.8 |
| **Q3 2020** | 16.7 (37) | 40.0 (21) | 30.8 (11) | 50.0 (18) |  |  |  |  |  |  |  | 31.5 |
| **Q4 2020** | 43.0 (35) | 16.7(11) | 30.0 (10) | 45.0 (20) |  |  |  |  |  |  |  | 18.2 |
| **Q1 2021** | 46.2 (52) | 38.1 (21) | 54.5 (11) | 44.4 (17) | 66.7 (12) | 47.6 (21) |  |  |  |  |  | 47.1 |
| **Q2 2021** | 36.4 (55) | 15.4 (26) | 7.1(14) | 60.0 (18) | 33.3 (15) | 44.4 (17) |  |  |  |  |  | 32.9 |
| **Q3 2021** | 18.9 (27) | 30.8 (26) | 30.8 (13) | 40.0 (4) | 27.3 (22) | 25.0 (19) |  |  |  |  |  | 26.0 |
| **Q4 2021** | 31.1 (41) | 29.4 (17) | 0 (11) | 27.8 (8) | 35.3 (16) | 28.1 (32) |  |  |  |  |  | 27.9 |
| **Q1 2022** | 18.9 (36) | 21.4 (14) | 25.0 (12) | 20.0 (4) | 23.1 (12) | 23.5 (34) | 9.1 (9) | 7.5 (36) | 34.0 (28) | 19.0 (20) | 13.5 (34) | 20.2 |
| **Q2 2022** | 40.0 (41) | 45.5 (10) | 26.7 (14) | 50.0 (7) | 11.1 (9) | 25.7 (35) | 28.6 (7) | 44.4 (10) | 41.4 (26) | 18.8 (15) | 8.8 (33) | 31.0 |
| **Q3 2022** | 33.3 (23) | 40.0 (10) | 20.0 (5) | 33.3 (8) | 40.0 (7) | 39.1 (21) | 66.7 (2) | 31.0 (29) | 35.0 (16) | 35.7 (28) | 24.1 (27) | 34.7 |
| **Q4 2022** | 35.0 (56) | 50.0 (8) | 75.0 (4) | 18.2 (9) | 37.1 (33) | 27.9 (19) | 50.0 (2) | 32.0 (25) | 33.3 (36) | 35.1 (53) | 38.2 (32) | 34.7 |

**Table S13: DKA duration in hours for hospitals A-K across timelines. n- number of DKA episodes.**

| **Timelines** | **A Median(IQR);n** | **B Median(IQR);n** | **C Median(IQR);n** | **D Median(IQR);n** | **E Median(IQR);n** | **F Median(IQR);n** | **G Median(IQR);n** | **H Median(IQR);n** | **I Median(IQR);n** | **J Median(IQR);n** | **K Median(IQR);n** |
| --- | --- | --- | --- | --- | --- | --- | --- | --- | --- | --- | --- |
| **Q1 2020** | 14.3 (9-19.2); 45 | 17.9 (14.7-39.2) ; 25 | 22.1 (15.8-66.8) ; 13 | 14.1 (9.1-21.4) ; 18 |  |  |  |  |  |  |  |
| **Q2 2020** | 10.6 (6.9-19) ; 36 | 28.5 (13.8-41) ; 19 | 24.9 (15.3-45.5) ; 15 | 15.5 (6.1-24.3) ; 16 |  |  |  |  |  |  |  |
| **Q3 2020** | 10.7 (8.4-20.7) ; 37 | 16.9 (10.2-22.1) ; 21 | 21 (14.5-25.1) ;11 | 13.7 (11.5-17.1) ; 18 |  |  |  |  |  |  |  |
| **Q4 2020** | 9 (5.8-12.9) ; 35 | 18.6 (13.4-25.5) ; 11 | 13.1 (7.7-21.5) ; 10 | 16.8 (10.5-28.1) ; 20 |  |  |  |  |  |  |  |
| **Q1 2021** | 13 (9-18.4) ; 52 | 21 (15.5-30.6) ; 21 | 21.9 (12.3-29.3) ; 11 | 15.2 (11.8-27.2) ; 17 | 9.1 (7.6-15.5); 12 | 11.7 (7.8-17.7) ; 21 |  |  |  |  |  |
| **Q2 2021** | 13.8 (9.3-20.3) ; 55 | 13.7 (10.9-20) ; 26 | 19.6 (14-22.9) ;14 | 15.3 (;8.9-27.3); 15 | 7.7 (5.4-10.6); 15 | 9.3 (6.6-14.8) ; 17 |  |  |  |  |  |
| **Q3 2021** | 12.8 (9.8-18.6) ; 27 | 16.2 (11.6-27.1) ; 26 | 17.6 (13.3-21.8) ; 13 | 10.6 (6.6-19.2) ; 4 | 10.5 (5.5-11.9) ; 22 | 15.3 (8.9-22) ; 19 |  |  |  |  |  |
| **Q4 2021** | 14.6 (10.2-20.5) ; 41 | 15.9 (12-23.4) ; 17 | 20.5 (15.4-45.7) ; 11 | 16 (6.8-24.6) ; 8 | 10.3 (7.3-17.8) ; 16 | 15.5 (8.3-28) ; 32 |  |  |  |  |  |
| **Q1 2022** | 12.6 (8.1-18.1) ; 36 | 11.5 (10.2-13.8) ; 14 | 14.7 (10.7-24.9) ; 12 | 11.1 (5.9-17.6) ; 4 | 13.8 (9.4-17.8) ; 12 | 19.6 (11.6-25.3) ; 34 | 11.0 (7.1 - 15.5); 9 | 17.9 (10.4 - 40.8); 40 | 20.5 (16.6 - 28.0); 28 | 10.7 (7.4 - 18.7); 20 | 34.0( 14.5-44.2); 34 |
| **Q2 2022** | 18.7 (12.9 - 29.2); 38 | 15.8 (8.4 - 27.0); 10 | 17.8 (9.1 - 22.6); 14 | 23.6 (17.9 - 28.3); 8 | 12.7 (6.6 - 14.7); 8 | 18.6 (13.0 - 31.8); 35 | 10.4 (7.3 - 15.2); 7 | 22.1 (10.8 - 26.1); 10 | 17.0 (9.9 - 32.6); 25 | 12.7 (7.6 - 16.6); 15 | 25.9 (16.3-44.1); 32 |
| **Q3 2022** | 15.1 (11.1 - 23.0); 23 | 20.1 (9.7 - 25.5); 10 | 14.7 (11.9 - 24.1); 5 | 16.2 (14.0 - 24.6); 8 | 13.8 (7.7 - 21.1); 7 | 18.5 (13.8 - 28.3); 21 | 9.1 (5.9 - N/A); 2 | 8.3 (6.4 - 12.2); 28 | 12.1 (7.3 - 15.3); 15 | 12.0 (9.4 - 19.4); 28 | 19.8( 31.2- 35.8); 24 |
| **Q4 2022** | 15.7 (11.8 - 30.6); 56 | 21.2 (11.4 - 30.8); 8 | 51.9 (13.1 - 88.5); 4 | 20.7 (16.0 - 26.3); 10 | 16.7 (13.0 - 21.0); 33 | 22.6(15.7-30.1);42 | 4.9 (4.9 - 4.9); 1 | 11.6 (8.2 - 14.6); 24 | 22.8 (17.8 - 32.4); 35 | 14.3 (8.3 - 24.6); 53 | 20.4 ( 14.6-28.4); 28 |

**Table S14: Length of stay in days for hospitals A-K across timelines. n- number of DKA episodes.**

| **Timelines** | **A Median(IQR);n** | **B Median(IQR);n** | **C Median(IQR);n** | **D Median(IQR);n** | **E Median(IQR);n** | **F Median(IQR);n** | **G Median(IQR);n** | **H Median(IQR);n** | **I Median(IQR);n** | **J Median(IQR);n** | **K Median(IQR);n** |
| --- | --- | --- | --- | --- | --- | --- | --- | --- | --- | --- | --- |
| **Q1 2020** | 3.2 (1.7-9.1) ; 45 | 2.5 (1.8-6.2) ; 25 | 3.3 (1.6-6.7) ; 13 | 4.8 (1.7-23.5) ; 18 |  |  |  |  |  |  |  |
| **Q2 2020** | 5.9 (2.2-12.1) ; 36 | 2.8 (1.7-8.9) ; 19 | 3.7 (2.3-5.1) ; 15 | 2.1 (1.5-3.8) ; 16 |  |  |  |  |  |  |  |
| **Q3 2020** | 3.1 (1.5-6) ; 37 | 2.4 (1.4-5.9) ; 21 | 3 (2.4-11) ; 11 | 2.6 (1.9-5.2) ; 18 |  |  |  |  |  |  |  |
| **Q4 2020** | 3.0 (1.6-5.8) ; 35 | 3.6 (2.6-5.3) ; 11 | 5.3 (3.9-7.8) ; 10 | 3 (1.8-7.7) ; 20 |  |  |  |  |  |  |  |
| **Q1 2021** | 4.6 (2.7-8.8) ; 52 | 3.8 (2.3- 7) ; 21 | 5.8 (3.6-16.7); 11 | 3.3 (1.4-7.3); 17 | 1.5 (1.1-4.9) ; 12 | 6 (3.6-8.5) ; 21 |  |  |  |  |  |
| **Q2 2021** | 5.2 (2.9-9.7) ; 55 | 4.1 (2.3-9.9) ; 26 | 4.8 (3.7-12.3) ; 14 | 5.7 (2.2-6) ; 15 | 3.1 (1-4.4) ; 15 | 5.2 (2.4-11.5) ; 17 |  |  |  |  |  |
| **Q3 2021** | 5.0 (2.9-13.3) ; 27 | 3.9 (1.9-5.6) ;26 | 2.6 (1.8-8.2) ; 13 | 10 (3.8-16.3) ;4 | 2.8 (1.2-4.2) ; 22 | 5.9 (3-14) ; 19 |  |  |  |  |  |
| **Q4 2021** | 3.4 (2.3-8.1); 41 | 2.4 (1.5-5.5) ; 17 | 2.2 (1.4-6.3) ; 11 | 2.5 (1.8-3.8) ; 8 | 2.5 (1.9-4) ; 16 | 4 (2.6-6.8) ; 32 |  |  |  |  |  |
| **Q1 2022** | 3.5 (2-4.9) ; 36 | 1.5 (1.1-2.7) ; 14 | 2.8 (2-6.7) ; 12 | 1.6 (0.8-6.3) ; 4 | 2.8 (1.9-3.1) ; 12 | 3.4 (2.2-6.4) ; 34 | 9.9 (8.0 - 179.3); 9 | 3.2 (1.9 - 5.3); 39 | 3.3 (2.1 - 9.7); 27 | 3.1 (1.8 - 5.0); 19 | 3.8 (2.8 - 10.3); 35 |
| **Q2 2022** | 3.3 (1.9 - 7.0); 38 | 1.8 (1.0 - 2.3); 9 | 2.4 (1.6 - 6.1); 13 | 4.5 (2.6 - 11.3); 8 | 2.6 (1.5 - 11.2); 9 | 3.6 (2.2 - 5.9); 32 | 6.9 (2.0 - 9.7); 7 | 1.8 (1.2 - 4.3); 9 | 3.4 (1.8 - 10.2); 26 | 4.0 (2.4 - 5.8); 14 | 4.3 (2.0 - 11.2); 33 |
| **Q3 2022** | 4.3 (3.1 - 9.4); 22 | 2.5 (2.0 - 12.0) | 3.0 (0.9 - 5.1); 5 | 3.2 (2.3 - 3.9); 8 | 4.6 (1.3 - 11.3); 8 | 3.2 (2.8 - 6.5); 19 | 2.6 (2.6 - 2.6); 1 | 2.8 (1.6 - 4.8); 26 | 2.1 (0.9 - 4.3); 17 | 5.2 (2.5 - 8.3); 27 | 3.0 (1.7 - 6.5); 26 |
| **Q4 2022** | 3.3 (2.3 - 5.7); 52 | 4.7 (2.4 - 7.9); 33 | 8.6 (6.8 - 9.0); 4 | 5.0 (2.2 - 7.8); 10 | 2.1 (1.2 - 2.9); 8 | 4.1(2.5-5.9); 42 | 1.1 (1.1 - 1.1); 1 | 3.0 (1.9 - 7.7); 24 | 5.4 (2.2 - 14.9); 32 | 3.5 (1.4 - 5.0); 49 | 2.6 (1.9 - 5.3); 32 |

**
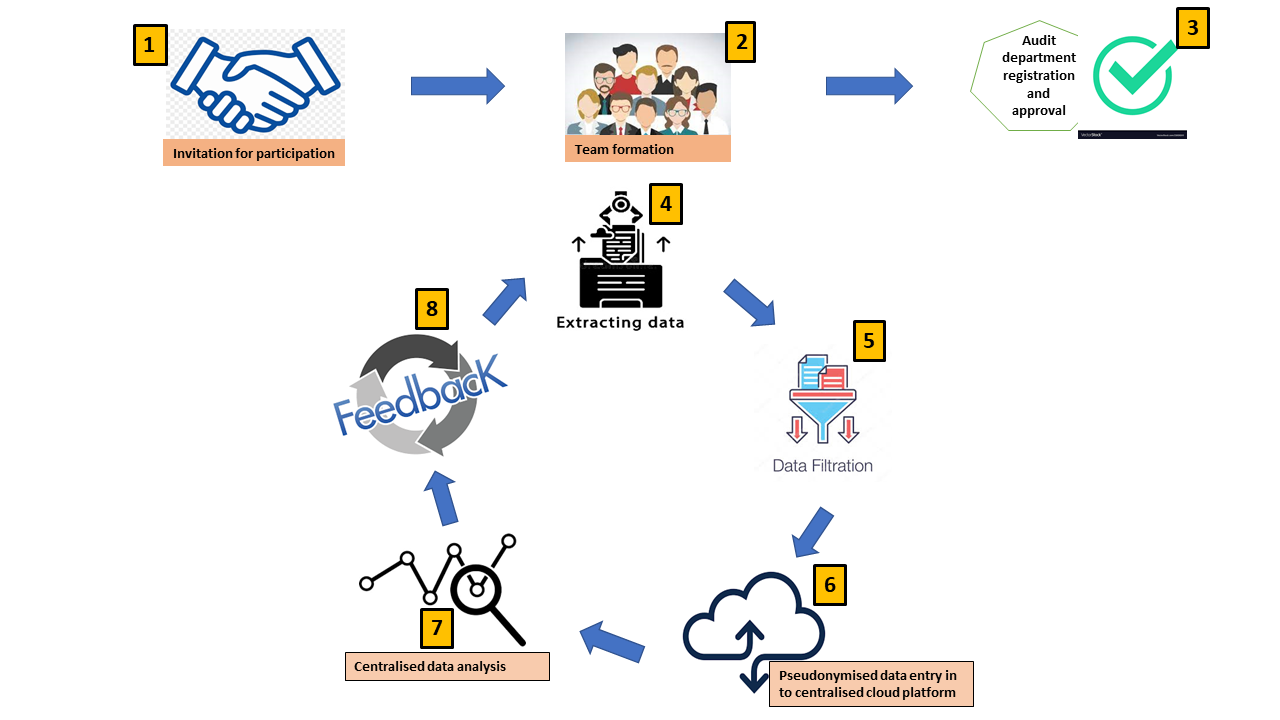
**

Figure S1: Process explaining the DEKODE working model across participating hospital


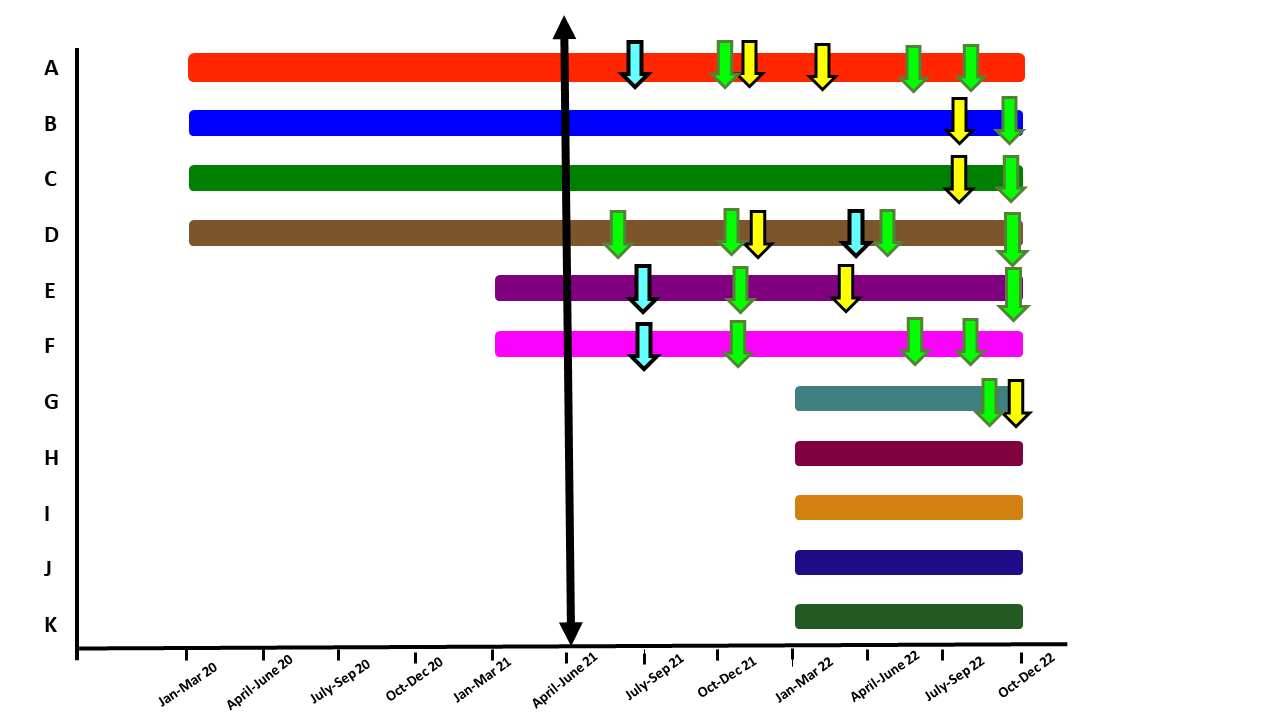


Figure S2: Timeline depicting interventions conducted across the ten hospitals. Black arrow- the timeline when JBDS introduced the guideline update of reducing FRIII to 0.05u/kg when glucose drops below 14mmol/L. Blue arrows- guideline changes that happened in respective hospitals. Green arrows- local non-digital interventions (local presentations to stakeholders, junior doctors, and nursing staff education on updated guidelines on DKA management). Yellow arrows- local digital interventions (circulation of updated guidelines through junior doctor WhatsApp groups, GIF circulation, bite-sized comic video circulation).
